# Steroidogenic Capacity of Ovarian Interstitial Tissue in the Koala (*Phascolarctos cinereus*): Morphological and Immunohistochemical Evidence

**DOI:** 10.3390/biology15010047

**Published:** 2025-12-27

**Authors:** Jackson Boyd, Stephen D. Johnston, Chiara Palmieri

**Affiliations:** 1 School of Environment, The University of Queensland, Gatton 4343, Australia; s.johnston1@uq.edu.au; 2 School of Veterinary Science, The University of Queensland, Gatton 4343, Australia; c.palmieri@uq.edu.au

**Keywords:** koala, interstitial tissue, steroidogenesis, ovary, luteinisation, immunohistochemistry, histology

## Abstract

Ovarian interstitial tissue (IT) is a prominent but poorly characterised component of the koala ovary. This study analysed the morphology and immunohistochemical profile of IT in the koala ovary across different reproductive phases. Ovaries from ten sexually mature females were examined histologically and immunolabelled for aromatase, HSD3B2, HSD17B1, the follicle stimulating hormone receptor (FSHR), and the luteinizing hormone receptor (LHR). IT occurred as multifocal cortical aggregates composed of two distinct cell types. Large interstitial cells were polygonal with highly vacuolated and weakly eosinophilic cytoplasm and were morphologically consistent with a steroidogenic phenotype. Small interstitial cells were densely eosinophilic, non-vacuolated, and morphologically uniform across reproductive phases. Immunohistochemical analysis revealed that small interstitial cells exhibited the broadest expression of steroidogenic markers in the ovary, with coexpression of HSD3B2, HSD17B1, aromatase, FSHR, and LHR. In contrast, large interstitial cells exhibited comparatively limited enzyme and receptor expression. Staining intensity across all markers was strongest during the interoestrous phase, moderate in the proliferative phase, reduced in the luteal phase, and minimal or absent when the koalas were lactating, suggesting potential cyclical variation in IT function. Granulosa, theca and germinal epithelial cells showed variable expression of steroidogenic enzymes and gonadotropin receptors, indicating that ovarian steroidogenesis in the koala may be more heterogeneous than predicted by the classical two-cell, two-gonadotropin model. Together, these findings provide preliminary evidence for the steroidogenic capacity of ovarian IT in the koala, indicating that its activity varies across reproductive phases, and appear to suggest a unique cellular organisation compared to other mammals. As these results are based on morphology and immunohistochemistry alone, further functional studies are required to confirm the steroidogenic output and clarify the physiological significance of IT in this species.

## Simple Summary

The koala ovary contains an unusually large amount of interstitial tissue (IT), the function of which is not well understood in this species. This study examined koala ovaries to identify the types of cells present in this tissue and to assess whether they express markers associated with the production of steroid hormones, which are important for regulating reproduction. By studying ovarian tissue from different reproductive phases, we found that koala IT consisted of two distinct cell types: one with the typical appearance of hormone-producing cells, and the second, smaller cell type, with the strongest evidence for the presence of key gonadotropin receptors and enzymes needed to produce steroid hormones. These signals were highest when the females were not in an active breeding phase and lowest during lactation, suggesting that the marker expression in this tissue changes across the reproductive cycle. These findings improve our understanding of koala reproductive biology and may assist future research aimed at supporting the conservation and management of this endangered marsupial.

## 1. Introduction

The koala (*Phascolarctos cinereus*) is an endangered arboreal herbivorous marsupial endemic to Australia [1,2]. While there has been a significant increase in our understanding of koala reproductive biology and in the application of reproductive technology over the last 25 years (see reviews by Johnston and Holt [3,4]), there are still significant gaps in our understanding of ovarian physiology. One of the most striking features of the ovary of this induced ovulating species is the abundance of interstitial tissue (IT) [5,6]. On histology, IT appears as clusters of cells forming discrete foci that often occupy large portions of the ovarian stroma [5]. Early anatomical studies also noted its presence but did not resolve its functional significance or origins [7]. Johnston [8] and Pagliarani [9] both identified two morphologically distinct interstitial cell types within each foci and proposed that hyperplasia of the theca interna and granulosa cells of atretic follicles gives rise to the tissue. The most recent synthesis of these findings emphasised that while IT is clearly prominent in the koala ovary, its precise cellular origin and function remain unresolved [5].

Morphological observations of the koala ovary have suggested that IT may be steroidogenic [5]; in particular, lipid droplets within interstitial cells resemble the steroidogenic phenotype exhibited in other endocrine tissues [5]. Johnston [8] hypothesised that IT may synthesise oestrogens that support pre-ovulatory follicle development. Pagliarani [9] reported strong immunoreactivity for 3β-hydroxysteroid dehydrogenase/Δ5→4-isomerase type 1 (HSD3B1) in both IT and corpora lutea, providing the first molecular evidence that koala IT may be capable of contributing to progestogen or androgen precursor synthesis. However, it remains unknown whether the interstitial foci express additional enzymes required for steroid synthesis or receptors enabling responsiveness to gonadotropins [9].

Comparative studies highlight the diversity of ovarian IT across mammals [10,11]. In many eutherians, IT may arise from theca cells of atretic follicles, as in cats [12], rabbits [13], guinea pigs [13], camels [14], and humans [15]. In marsupials, however, IT can have different origins including the medullary cords in brushtail possums (*Trichosurus vulpecula*) [16,17] and the rete cords in tammar wallabies (*Notamacropus eugenii)* [18]. This variation complicates functional assumptions and underscores the need for species-specific analyses [11]. The koala, therefore, represents an important model for understanding both the conservation of ovarian cellular architecture across mammals and the evolutionary diversity of steroidogenic tissue in marsupials.

The rabbit ovary contains abundant IT that appears to secrete 20α-dihydroprogesterone, a progestogen metabolite that can be reconverted to progesterone [19]. Prominent IT in ovaries of other species, including the southern hairy-nosed wombat (*Lasiorhinus latifrons*) [20], brushtail possum [16,17], and Asiatic yellow bat (*Scotophilus heathii*) [21] has also been characterised as steroidogenic based on morphology, expression of steroidogenic enzyme mRNA, and enzyme immunolocalisation, respectively.

Steroidogenic capacity can be inferred through the expression of key enzymes and receptors. Aromatase (CYP19A1) converts androgens to estrogens [22], 3β-hydroxysteroid dehydrogenase/Δ5→4-isomerase type 2 (HSD3B2) catalyses progestogen and androgen synthesis [23], and 17β-hydroxysteroid dehydrogenase type 1 (HSD17B1) regulates the interconversion of estrone and oestradiol [24]. The follicle-stimulating hormone receptor (FSHR) and luteinizing hormone receptor (LHR) mediate gonadotropin responsiveness, typically localising to granulosa cell and to theca or luteal cell membranes, respectively [25]. Whether koala IT expresses these receptors, indicative of gonadotropin responsiveness, has not previously been addressed.

Koalas undergo complex reproductive cycles, including an interoestrous period if not mated, as well as proliferative, luteal, and lactational anoestrous phases [26]. Interstitial foci may vary in prevalence or morphology depending on these phases, as suggested by Johnston [8] who concluded that repeated anovulatory cycles could contribute to IT abundance. No study has systematically described IT morphology or immunophenotype across these defined reproductive phases. As ovarian steroidogenic activity and gonadotropin responsiveness are tightly regulated across the reproductive cycle, the absence of information on whether interstitial foci exhibit cycle-dependent variation further limits functional interpretation of this tissue [26].

The aim of this study was to characterise the morphology and immunohistochemical localisation of steroidogenic enzymes and gonadotropin receptors in koala ovarian interstitial foci across reproductive phases. While immunohistochemistry does not provide direct evidence of steroid output, it does allow strong inference of steroidogenic capacity and gonadotropin responsiveness [27]. We hypothesised that ovarian IT in the koala exhibits steroidogenic properties, reflected by its morphology and the expression and localisation of key enzymes and receptors, and that these features vary across reproductive phases. By providing preliminary molecular and morphological evidence, this study seeks to clarify the potential role of IT in koala ovarian physiology and to contribute to the broader understanding of marsupial reproductive biology.

## 2. Materials and Methods

### 2.1. Animals and Tissue Collection

Reproductive tissues were sourced from three previously archived samples and seven freshly performed koala necropsies. Formalin-fixed paraffin-embedded (FFPE) blocks containing the ovaries of koalas from the dataset of Pagliarani [9] (n = 3) were accessed with the authors’ approval. These tissues were collected during 2018 and 2019, were fixed in 10% neutral-buffered formalin (NBF) for 48 h immediately post-dissection and stored in 70% ethanol. Fresh samples were opportunistically collected in 2023 from sexually mature female koalas admitted to Currumbin Wildlife Hospital (n = 1) (CWH, Gold Coast, Queensland) and RSPCA Wildlife Hospital (n = 6) (Wacol, Queensland). All animals were euthanised for animal welfare reasons unrelated to the current study and clinical conditions were determined as independent of ovarian structure and function [9,28]. As a result, sample collection was opportunistic, which may introduce selection bias and variability related to animal condition and reproductive status. Sample collection and use was approved by the University of Queensland Animal Ethics Committee (ANFRA/UQ Approval 2021/AE001080). As samples were obtained from post-mortem clinical cases, no additional scientific collection permits were required. Necropsies were performed within two hours of euthanasia. The reproductive tract, including uteri, ovaries, oviducts, and ovarian bursae, was excised together. Bursae were reflected to expose the ovaries, and excess connective tissue was removed. Tissues were fixed in 10% NBF for 48 h and then transferred to 70% ethanol for storage.

### 2.2. Histological Processing

Ovaries were bisected sagittally to reveal internal structures, and both halves were included. 5 mm transverse sections of the uteri were prepared to help verify the phase of the reproductive cycle. Samples were processed overnight using a vacuum infiltration processor (Tissue-Tek VIP^®^ 6 AI; Sakura Finetek Japan Co., Ltd., Tokyo, Japan), embedded in paraffin, and sectioned at 4 µm on an automated rotary microtome (RM2255; Leica Biosystems GmbH, Nussloch, Germany). Haematoxylin and eosin (H&E) staining was performed on an autostainer (ST5020; Leica Biosystems GmbH, Nussloch, Germany), and slides were mounted with coverslips. Unstained sections were reserved for immunohistochemistry.

### 2.3. Phase of the Reproductive Cycle

Phases of the koala reproductive cycle were classified as (i) interoestrus, (ii) proliferative, or (iii) luteal based on ovarian and uterine morphology following definitions previously described by Johnston [8] and Pagliarani [9] (Table 1). No concurrent endocrine data were available for these animals, and reproductive phase classification was therefore based on morphological criteria alone. Females presenting with a pouch young and/or lactating mammary glands were presumed and classified as being in lactational anoestrus, however we also recognised that the duration of lactation in the koala may result in inter-individual variation in ovarian morphology and endocrine status [29]. In total, the ovaries (n = 20) from ten females were utilised in this study. Of these animals, four were classified as being in a period of interoestrus, two were in the proliferative phase, two were in the luteal phase, and two were in lactational anoestrus.

### 2.4. Immunohistochemistry

Primary antibodies were selected based on sequence homology with koala proteins. Candidate antibodies were assessed using the NCBI protein basic local alignment search tool (BLASTp; BLAST+ suite, National Center for Biotechnology Information, Bethesda MD, USA) for protein-to-protein sequence comparison against the non-redundant protein (nr) database to confirm a high degree of predicted identity with koala target sequences prior to inclusion. A minimum threshold of 60% sequence homology was utilised for antibody selection, as this level of conservation is generally considered sufficient to support cross-reactivity of polyclonal antibodies across mammalian species when combined with appropriate histological localisation and internal control validation [30,31,32]. The antibodies used in this study were anti-aromatase (PA5-109235; Invitrogen, Shanghai, China), anti-HSD3B2 (PA5-106895; Invitrogen, Shanghai, China), anti-HSD17B1 (PA5-42058; Invitrogen, Shanghai, China), anti-LHR (PA5-79598; Invitrogen, Shanghai, China), and anti-FSHR (PA5-99424; Invitrogen, Shanghai, China) (see Table 2).

Immunohistochemistry was performed on 4 µm sections of FFPE koala ovarian tissue using the Ventana Discovery Ultra automated staining platform (Ventana Medical Systems, Inc., Tucson, AZ, USA). To minimise technical variability when comparing staining intensity across reproductive phases, all sections were processed using identical staining conditions, antigen retrieval protocols, and detection chemistry, with each antibody applied at its optimised dilution consistently across all samples and controls. Negative controls included (i) omission of primary antibody on koala ovarian sections, and (ii) koala colon for anti-LHR, koala lung for anti-FSHR, anti-aromatase and anti-HSD3B2, and koala skeletal muscle for anti-HSD17B1, which do not express the target proteins. All controls were processed in parallel under identical conditions. Slides were dried at 60 °C for 8 min, deparaffinised on-instrument using EZ Prep solution (Ventana Medical Systems, Inc., Tucson, AZ, USA), and subjected to heat-induced, low pH (6.0) antigen retrieval in cell conditioning 2 buffer (CC2; Ventana Medical Systems, Inc., Tucson, AZ, USA) at high temperature (~95 °C) for 32 min. Endogenous peroxidase activity was blocked with Inhibitor CM (Ventana Medical Systems, Inc., Tucson, AZ, USA) for 12 min. Each primary antibody was manually applied at its optimised dilution and incubated at room temperature for 60 min. Antibody binding was detected using a horse radish peroxidase (HRP)-conjugated anti-rabbit multimer (Ventana Medical Systems, Inc., Tucson, AZ, USA), which served as both the secondary antibody and signal amplification system. Visualisation was achieved with 3,3′-diaminobenzidine (DAB; Ventana Medical Systems, Inc., Tucson, AZ, USA) as the chromogen. Sections were counterstained with haematoxylin for 8 min, treated with bluing reagent for 4 min, dehydrated through graded ethanol, cleared in xylene, and mounted with coverslips. Ovarian sections were then photographed using a slide scanner (Slideview VS200; Olympus Corporation, Tokyo, Japan) and images were viewed using the associated scanner software (OlyVIA V4.2; Evident Scientific, Tokyo, Japan).

Staining intensity for each antibody and cell type was assessed semi-quantitatively across all ovarian samples. Each tissue section was examined independently by a single observer, blinded to reproductive phase, and staining strength was classified as negative, mild, moderate, or strong (−, +, ++, +++). Classifications were assigned based on the predominant intensity observed within each cell population for each whole ovary.

## 3. Results

### 3.1. Interstitial Cell Morphology

**Figure 1:**
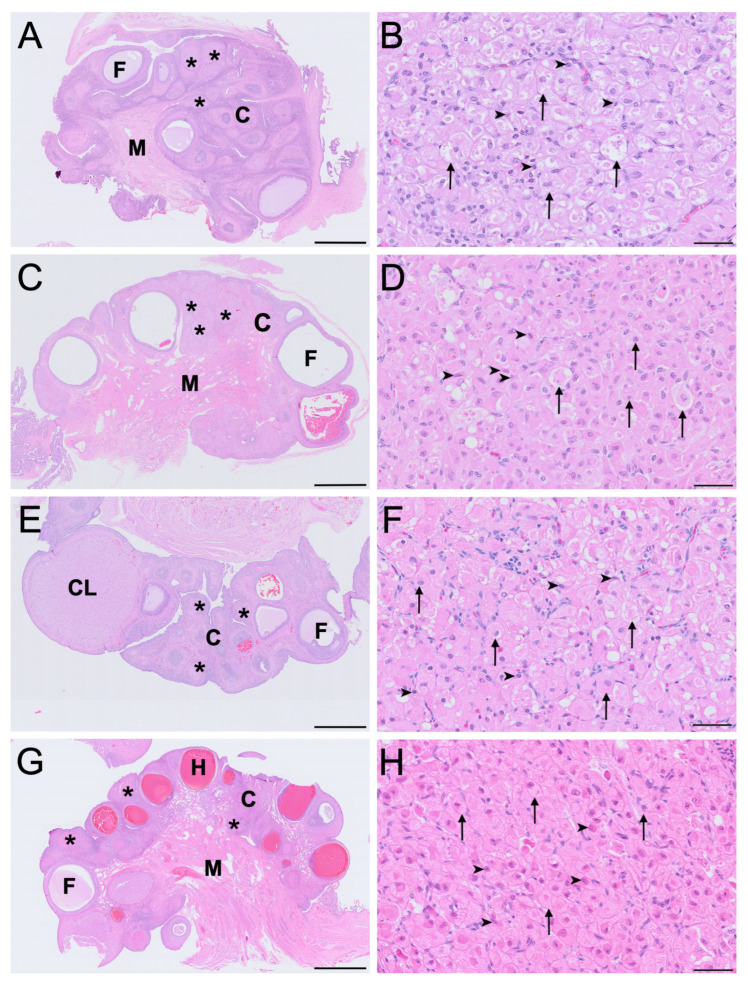
Histological features of koala ovary (sagittal section (**A**,**C**,**E**,**G)**) and ovarian interstitial tissue (**B**,**D**,**F**,**H**) during the interoestrous (**A**,**B**), proliferative (**C**,**D**), luteal (**E**,**F**) and lactational anoestrous (**G**,**H**) phases, stained with H&E. High-magnification panels (**B**,**D**,**F**,**H**) are representative of interstitial tissue morphology at each reproductive phase. C = cortex; CL = corpus luteum; F = follicle; H = haemorrhagic follicle; M = medulla; asterisks = interstitial tissue foci; arrowheads = small interstitial cells; arrows = large interstitial cells. Bar = 1 mm (**A**,**C**,**E**,**G**); Bar = 50 μm (**B**,**D**,**F**,**H**).

IT was abundant in ovaries across all reproductive phases, forming spherical, multi-focal aggregates often occupying the majority of the interfollicular cortical space, as shown in Figure 1. These foci were composed of two morphologically distinct cell populations–large interstitial cells and small interstitial cells and were encapsulated by multilaminar fusiform and less commonly polygonal cells resembling stromal fibroblasts (Figure 1B,D,F,H). The mean ± SD diameter of large interstitial cells across reproductive phases was 28.0 ± 6.1 μm, while small interstitial cells averaged 9.3 ± 2.2 μm. The large interstitial cells were rounded to polygonal in shape, exhibiting a weakly eosinophilic cytoplasm and variable degrees of cytoplasmic vacuolation. In contrast, the small interstitial cells were polygonal to spindle-shaped with dense, eosinophilic cytoplasm and minimal vacuolation. The nuclei of large interstitial cells were typically round and euchromatic in appearance with loosely arranged chromatin, while the nuclei of small interstitial cells were flattened or irregular with densely compact chromatin. Although the small interstitial cells maintained a consistent morphology throughout the reproductive phases, large interstitial cells exhibited varying degrees of cytoplasmic vacuolation and eosinophilia.

In the interoestrous ovaries, the large interstitial cells were largest across the reproductive phases (mean ± SD = 32.4 ± 5.3 μm diameter). During this phase, these cells displayed extensive cytoplasmic vacuolation, appearing foamy, consistent with the presence of lipid inclusions lost during paraffin processing, and weak eosinophilia as shown in Figure 1B. In contrast, small interstitial cells measured 10.5 ± 1.4 μm in mean diameter, and were strongly eosinophilic, and non-vacuolated (Figure 1B). In ovaries of the proliferative phase, large interstitial cells (mean ± SD = 29.6 ± 5.2 μm diameter) exhibited mild to moderate vacuolation, indicative of reduced lipid content prior to processing, with more granular cytoplasm (Figure 1D). Small interstitial cells (mean ± SD = 10.6 ± 2.1 μm diameter) remained strongly eosinophilic with no vacuolation. IT in the luteal phase was composed of large interstitial cells that exhibited reduced size (mean ± SD = 24.8 ± 4.6 μm diameter), minimally vacuolated or non-vacuolated cytoplasm, increased cytoplasmic eosinophilia and occasional dense chromatin (Figure 1F). Small interstitial cells (mean ± SD = 7.0 ± 0.3 μm diameter) appeared smaller, compact, and strongly eosinophilic with dense nuclei during the luteal phase. In the ovaries of lactating koalas, large interstitial cells were similarly smaller in size (mean ± SD = 25.3 ± 4.9 μm diameter), non-vacuolated, and weakly eosinophilic with shrunken nuclei and condensed chromatin, whereas the small interstitial cells (mean ± SD = 9.2 ± 2.7 μm diameter) maintained a compact, strongly eosinophilic, non-vacuolated morphology (Figure 1H).

Notably, cytoplasmic vacuolation was most prominent in large interstitial cells during interoestrus and least evident in lactational anoestrus. The morphology of small interstitial cells remained relatively consistent across phases, with strong eosinophilia and dense chromatin but no observable vacuolation indicative of cytoplasmic lipid droplets (Figure 1B,D,F,H).

### 3.2. Immunohistochemistry

**Figure 2:**
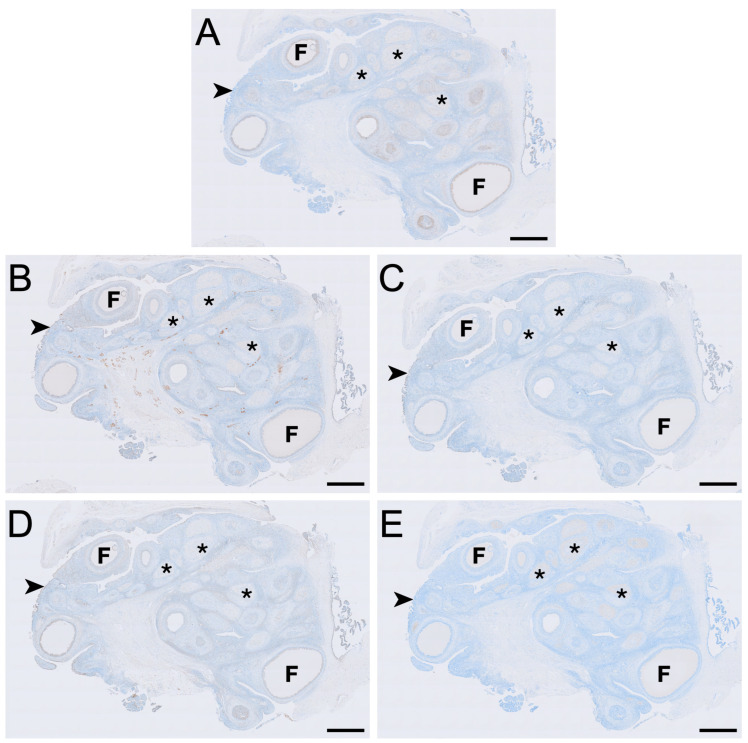
Immunolocalisation of LHR (**A**), FSHR (**B**), aromatase (**C**), HSD3B2 (**D**) and HSD17B1 (**E**) in a koala ovary during interoestrous phase. (**A**) LHR immunolocalisation showing staining in granulosa, theca externa and interstitial cells. (**B**) FSHR immunolocalisation showing staining in granulosa, theca interna, theca externa, interstitial, and germinal epithelial cells. (**C**) Aromatase immunolocalisation showing staining in granulosa, theca interna, theca externa, interstitial, and germinal epithelial cells. (**D**) HSD3B2 immunolocalisation showing staining in granulosa, theca interna, theca externa, interstitial, and germinal epithelial cells. (**E**) HSD17B1 immunolocalisation showing staining in interstitial cells. Immunopositive staining is visualised as brown precipitate (DAB), with nuclei counterstained blue with haematoxylin. F = follicles; asterisks = interstitial tissue foci; arrowheads = germinal epithelium. Bar = 1 mm.

The immunohistochemical LHR, FSHR, aromatase, HSD3B2 and HSD17B1 staining intensity for the ovarian cell types across the four reproductive phases of the koala are reported in Table 3. The modal classification for each cell type represents the most prominent staining intensity observed across ovaries in each reproducitve phase. For reproductive phases that were represented by an even number of ovaries, if two staining intensities were equally observed, both are represented as the modal classification, separated by a forward slash. Narrative descriptions of modal staining intensity are provided to aid interpretation but correspond directly to the semi-quantitative scores presented in Table 3. Figure 2 represents the distribution of staining for each cell type across a whole ovary during the interoestrous phase. Unless otherwise stated, histological images presented are representative of the modal staining patterns and morphological features observed consistently across all animals examined within each reproductive phase.

#### 3.2.1. Luteinising Hormone Receptor

**Figure 3:**
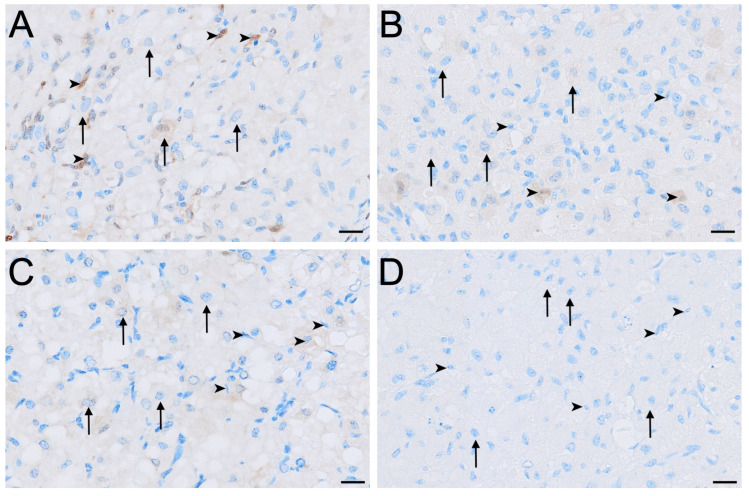
Immunolocalisation of LHR in interstitial cells of the koala ovary during interoestrous (**A**), proliferative (**B**), luteal (**C**) and lactational anoestrous (**D**) phases. (**A**) Interstitial cells during interoestrous phase showing moderate staining in small interstitial cells and mild staining in large interstitial cells. (**B**) Interstitial cells during proliferative phase showing mild to moderate staining in small interstitial cells and mild staining in large interstitial cells. (**C**) Interstitial cells during luteal phase showing mild to moderate staining in small interstitial cells and negative to mild staining in large interstitial cells. (**D**) Interstitial cells during lactational anoestrous phase showing negative staining in both small and large interstitial cells. Immunopositive staining is visualised as brown precipitate (DAB), with nuclei counterstained blue with haematoxylin. Arrowheads = small interstitial cells; arrows = large interstitial cells. Bar = 50 μm.

**Figure 4:**
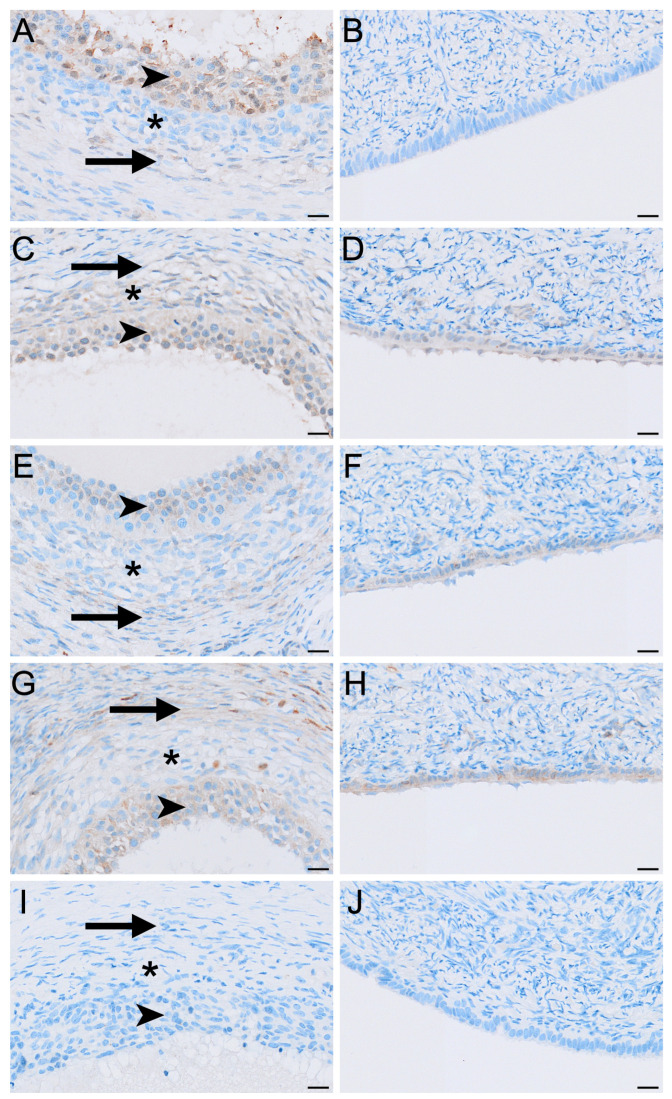
Immunolocalisation of LHR (**A**,**B**), FSHR (**C**,**D**), aromatase (**E**,**F**), HSD3B2 (**G**,**H**) and HSD17B1 (**I**,**J**) in granulosa and theca layers (**A**,**C**,**E**,**G**,**I**) and the germinal epithelium (**B**,**D**,**F**,**H**,**J**) in the koala ovary during interoestrous phase. (**A**) Moderate staining in granulosa cells, negative to mild staining in the theca interna, and negative to mild staining in the theca externa. (**B**) Negative staining in the germinal epithelium. (**C**) Mild to moderate staining in granulosa cells, mild staining in the theca interna, and mild staining in the theca externa. (**D**) Mild staining in the germinal epithelium. (**E**) Mild staining in granulosa cells, negative to mild staining in the theca interna, and mild staining in the theca externa. (**F**) Mild staining in the germinal epithelium. (**G**) Moderate staining in granulosa cells, mild staining in the theca interna, and mild to moderate staining in the theca externa. (**H**) Moderate staining in the germinal epithelium. (**I**) Negative staining in granulosa, theca interna, and theca externa. (**J**) Negative staining in the germinal epithelium. Immunopositive staining is visualised as brown precipitate (DAB), with nuclei counterstained blue with haematoxylin. Arrowheads = granulosa cells; asterisks = theca interna cells; arrows = theca externa cells. Bar = 20 μm.

LHR immunolabelling was present in all reproductive phases but varied in intensity among cell types and phases (Figure 2, Figure 3 and Figure 4). The strongest LHR immunoreactivity occurred in granulosa (Figure 2A and Figure 4A) and small interstitial cells (Figure 3A) during interoestrus, where staining was recorded as moderate. Similarly, small interstitial cells exhibited mild to moderate staining in the proliferative (Figure 3B) and luteal (Figure 3C) phases but were negative during lactational anoestrus (Figure 3D). Large interstitial cells were mildly positive during the interoestrous (Figure 3A) and proliferative (Figure 3B) phases but displayed negative to mild staining in the luteal phase (Figure 3C) and were negative during lactation (Figure 3D). Granulosa cells showed moderate staining in interoestrus (Figure 4A), mild to moderate staining in the proliferative phase, were negative to moderate in the luteal phase, and mild in lactational anoestrus. Theca interna and externa were negative to mild across reproductive phases (Figure 4A), and germinal epithelium remained negative throughout all phases (Figure 4B). Both negative control tissues were negative (Figure S1A,B).

#### 3.2.2. Follicle-Stimulating Hormone Receptor

**Figure 5:**
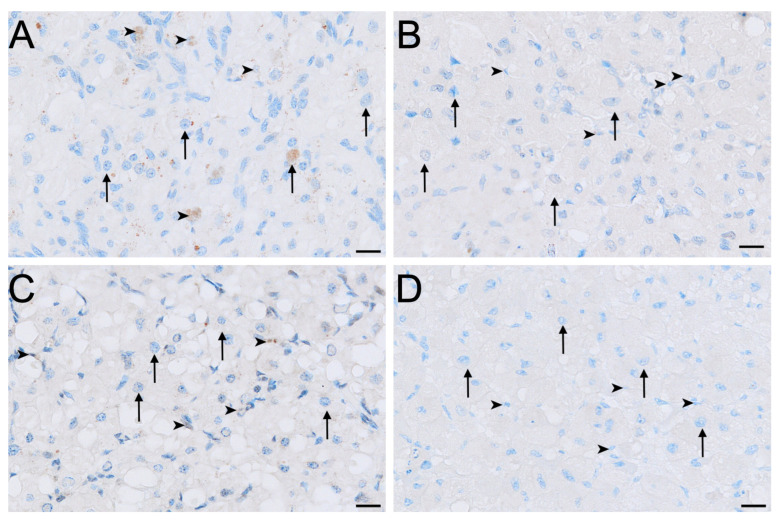
Immunolocalisation of FSHR in interstitial cells of the koala ovary during interoestrous (**A**), proliferative (**B**), luteal (**C**) and lactational anoestrous (**D**) phases. (**A**) Interstitial cells during interoestrous phase showing strong staining in small interstitial cells and mild staining in large interstitial cells. (**B**) Interstitial cells during proliferative phase showing mild staining in small interstitial cells and mild staining in large interstitial cells. (**C**) Interstitial cells during luteal phase showing negative to mild staining in both small and large interstitial cells. (**D**) Interstitial cells during lactational anoestrous phase showing mild staining in both small and large interstitial cells. Immunopositive staining is visualised as brown precipitate (DAB), with nuclei counterstained blue with haematoxylin. Arrowheads = small interstitial cells; arrows = large interstitial cells. Bar = 50 μm.

FSHR immunoreactivity was detected in all ovarian cell types and phases analysed except for the germinal epithelium of lactational ovaries (Figure 2, Figure 4 and Figure 5). Ovaries in interoestrous and proliferative phases exhibited the strongest staining intensity across cell types, while those classified as luteal or lactational were comparatively weaker (Figure 5). Small interstitial cells exhibited the highest staining intensity during interoestrus, where strong immunolocalisation was observed (Figure 5A). Small interstitial cells showed mild staining in ovaries during the proliferative phase, negative to mild in the luteal phase, and mild in lactational anoestrus (Figure 5B–D). Large interstitial cells showed mild staining in ovaries during interoestrous and proliferative phases and were negative to mild in luteal and mild in lactational ovaries (Figure 5A–D). Granulosa cells were mild to moderate in interoestrus (Figure 4C), mild in ovaries during the proliferative phase, negative to mild in luteal and mild in lactational ovaries. Theca interna cells were mild across reproductive phases, though were negative to mild in luteal ovaries (Figure 4C). Theca externa cells stained mildly in ovaries during the interoestrous (Figure 4C) and proliferative phases, negative to mild in luteal and mild to moderate in lactational samples. Germinal epithelial cells showed moderate staining during interoestrus (Figure 4D), mild staining in ovaries during the proliferative phase, negative to mild staining in ovaries during the luteal phase, and negative staining during lactational anoestrus. Both negative control tissues were negative (Figure S1C,D).

#### 3.2.3. Aromatase

**Figure 6:**
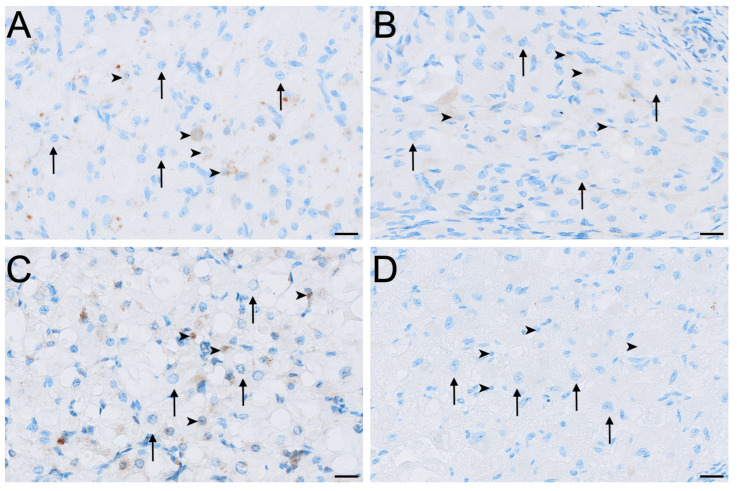
Immunolocalisation of aromatase in interstitial cells of the koala ovary during interoestrous (**A**), proliferative (**B**), luteal (**C**) and lactational anoestrous (**D**) phases. (**A**) Interstitial cells during interoestrous phase showing moderate staining in small interstitial cells and mild staining in large interstitial cells. (**B**) Interstitial cells during proliferative phase showing mild staining in small interstitial cells and negative staining in large interstitial cells. (**C**) Interstitial cells during luteal phase showing mild to moderate staining in small interstitial cells and negative to mild staining in large interstitial cells. (**D**) Interstitial cells during lactational anoestrous phase showing negative staining in both small and large interstitial cells. Immunopositive staining is visualised as brown precipitate (DAB), with nuclei counterstained blue with haematoxylin. Arrowheads = small interstitial cells; arrows = large interstitial cells. Bar = 50 μm.

Aromatase immunolabelling varied across both cell type and reproductive phase (Figure 4E,F and Figure 6). Immunolocalisation of aromatase in IT was strongest during interoestrus, with large interstitial cells displaying mild staining, while staining in small interstitial cells was observed as moderate (Figure 2C and Figure 6A). All ovaries in the proliferative phase contained large interstitial cells that were negative, and small interstitial cells that were mildly stained (Figure 6B). Ovaries in the luteal phase possessed large interstitial cells that were negative to mild, whereas the small interstitial cells were mild to moderate; both cell types were negative in the ovaries of lactating koalas (Figure 6C,D). Granulosa cells were mild in the interoestrous (Figure 4E) and proliferative phases, mild to moderate in the luteal phase, and mild in lactating koalas. Theca interna cells were negative to mild during the interoestrous (Figure 4E) and proliferative phases, mild in the luteal phase, and negative in lactational anoestrus. Theca externa cells were mild in the interoestrous (Figure 4E) and luteal phases, negative in the proliferative phase and in koalas that were lactating. The germinal epithelium stained mildly in the interoestrous (Figure 4F) and proliferative phases, mild to moderate in the luteal phases, and negative to mild during lactation. Both negative control tissues were negative (Figure S1E,F).

#### 3.2.4. HSD3B2

**Figure 7:**
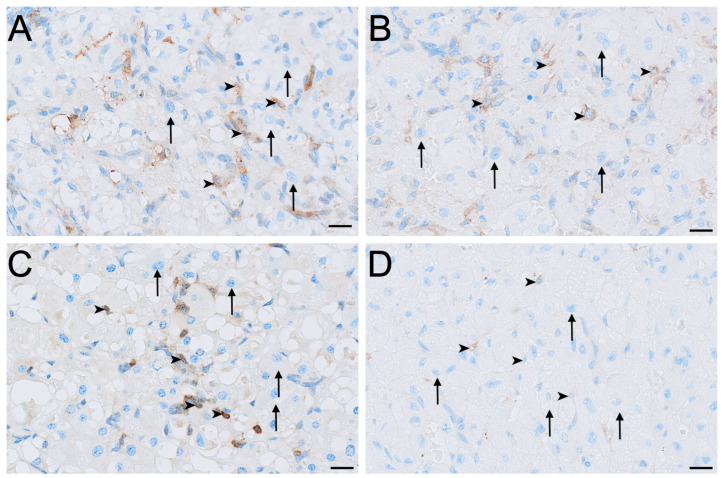
Immunolocalisation of HSD3B2 in interstitial cells of the koala ovary during interoestrous (**A**), proliferative (**B**), luteal (**C**) and lactational anoestrous (**D**) phases. (**A**) Interstitial cells during interoestrous phase showing moderate to strong staining in small interstitial cells and mild staining in large interstitial cells. (**B**) Interstitial cells during proliferative phase showing moderate to strong staining in small interstitial cells and mild staining in large interstitial cells. (**C**) Interstitial cells during luteal phase showing moderate to strong staining in small interstitial cells and mild staining in large interstitial cells. (**D**) Interstitial cells during lactational anoestrous phase showing mild staining in small interstitial cells and negative staining in large interstitial cells. Immunopositive staining is visualised as brown precipitate (DAB), with nuclei counterstained blue with haematoxylin. Arrowheads = small interstitial cells; arrows = large interstitial cells. Bar = 50 μm.

HSD3B2 immunolocalisation was consistently strongest in the small interstitial cells, while large interstitial cells stained contrastingly milder in comparison (Figure 7). Small interstitial cells exhibited moderate to strong staining for HSD3B2 in ovaries in the interoestrous, proliferative and luteal phases, but stained mildly during lactational anoestrus (Figure 7). Large interstitial cells displayed a similar pattern, showing mild staining during the interoestrous, proliferative, and luteal phases, becoming negative in lactation (Figure 7). Granulosa cells stained moderately in interoestrus (Figure 4G), mild in the proliferative phases, mild to moderate in the luteal phase, and mild in lactating koalas. The theca interna showed mild staining in interoestrus (Figure 4G), negative to mild staining in the proliferative and luteal phases, and negative to moderate staining in lactation. Theca externa cells exhibited mild to moderate staining across interoestrous, proliferative and luteal phases (Figure 4G) and moderate in lactating koalas. The germinal epithelium was moderate in interoestrus (Figure 4H), mild in the proliferative phase, mild to moderate in luteal phase, and mild in lactational anoestrus. Both negative control tissues were negative (Figure S1G,H).

#### 3.2.5. HSD17B1

**Figure 8:**
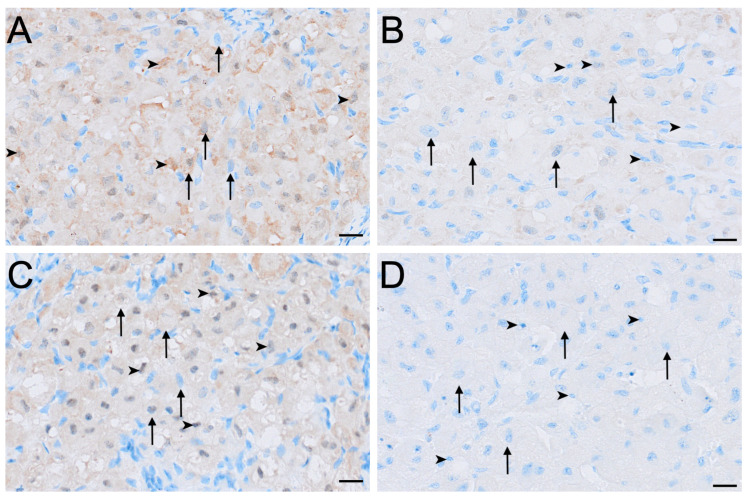
Immunolocalisation of HSD17B1 in interstitial cells of the koala ovary during interoestrous (**A**), proliferative (**B**), luteal (**C**) and lactational anoestrous (**D**) phases. (**A**) Interstitial cells during interoestrous phase showing strong staining in small interstitial cells and moderate staining in large interstitial cells. (**B**) Interstitial cells during proliferative phase showing moderate staining in small interstitial cells and mild to moderate staining in large interstitial cells. (**C**) Interstitial cells during luteal phase showing negative to moderate staining in both small and large interstitial cells. (**D**) Interstitial cells during lactational anoestrous phase showing mild staining in both small and large interstitial cells. Immunopositive staining is visualised as brown precipitate (DAB), with nuclei counterstained blue with haematoxylin. Arrowheads = small interstitial cells; arrows = large interstitial cells. Bar = 50 μm.

HSD17B1 was expressed exclusively in the interstitial cells in all ovaries (Figure 2E). Small interstitial cells exhibited strong staining in interoestrus, moderate staining in ovaries in the proliferative phase, were negative to moderate in luteal ovaries, and were mild in lactational anoestrus (Figure 8). Staining in large interstitial cells was moderate in interoestrus and mild to moderate in proliferative ovaries, negative to moderate in luteal ovaries, and mild in lactational anoestrus (Figure 8). Granulosa, theca, and germinal epithelial cells were consistently negative across reproductive phases (Figure 4I,J). Both negative control tissues were negative (Figure S1I,J).

## 4. Discussion

The morphology and immunohistochemical profile of IT in the koala ovary suggest that it possesses the molecular components necessary for and therefore may be capable of steroidogenesis. We suggest that the results of this study provide preliminary evidence that IT possesses steroidogenic capacity and that variation in marker expression may be associated with the phase of the reproductive cycle. IT was present in all ovarian sections and was characterised by circular foci composed of two distinct cell types, consistent with the description of Pagliarani et al. [5]. The morphology of the large interstitial cells was consistent with the classical luteinised and steroidogenic phenotype of interstitial glands described in other species [10,11]. Given that routine paraffin processing removes neutral lipids, the cytoplasmic vacuolation observed in these cells is most likely to be lipid droplets. The appearance of these vacuoles is consistent with dissolved lipid inclusions which are interpreted as cholesterol-ester stores that serve as precursors for steroid synthesis in other species [33], including the cat [12], rabbit [34] and wombat [20]. Euchromatic nuclei in these cells are indicative of active transcription [35], which has also been described as a characteristic of the steroidogenic phenotype in other species [36,37]. In a review by Guraya [37], the cells of mammalian ovarian interstitial glands were characterised as large, polygonal and highly vacuolated. The cytoplasm of these cells was described as containing abundant lipid inclusions composed of phospholipids, triglycerides and cholesterol esters, together with prominent smooth endoplasmic reticulum and pleomorphic mitochondria with tubular cristae [37]. These features, which differentiate interstitial cells from the more compressed stromal elements of the ovarian cortex, correspond closely to the vacuolated, lipid-laden appearance of the large interstitial cells observed in the koala ovary.

Across reproductive phases, the most consistent morphological change in large interstitial cells was the degree of cytoplasmic vacuolation, which was greatest in the interoestrous phase and progressively diminished through the proliferative and luteal phases, becoming minimal in lactating koalas. We speculate that this cyclical change may reflect phase-associated mobilisation of lipid precursors in support of steroidogenic capacity. In the plains viscacha (*Lagostomus maximus maximus*), cytoplasmic lipid droplets in interstitial cells were prominent prior to luteal activation, however, became notably reduced in size and number during pregnancy [36]. The authors interpreted this change as a shift from pre-pregnancy accumulation and storage to mobilisation and use in support of the increased endocrine demands of pregnancy [36]. A comparable pattern may be present in the koala, where vacuolation of large interstitial cells is greatest in interoestrus and diminishes through proliferative, luteal and lactational phases. Although lipid identity cannot be confirmed in paraffin-embedded tissue, this reduction may likewise reflect phase-dependent mobilisation of stored substrates.

In contrast, the small interstitial cell type in the koala ovary lacked the characteristics that typify both IT and steroidogenic cells in other species. Small interstitial cells were morphologically distinct from large interstitial cells, exhibiting polygonal to spindle-shaped profiles, a non-vacuolated and strongly eosinophilic cytoplasm, and round to flattened nuclei with densely compacted chromatin. While the small sample size of this study limits the scope of interpretation, the small interstitial cells appeared to maintain a consistent morphology throughout the reproductive phases, potentially indicating minimal structural responsiveness to differing endocrine states. Based on morphology alone, the large interstitial cell type appears to exhibit features consistent with a greater steroidogenic capacity, while the small interstitial cell type displays a less differentiated morphology that could reflect an alternative or earlier cellular state, although developmental relationships cannot be inferred from the present data [11].

The heterogeneous structure of interstitial foci in the koala ovary is apparently unique amongst mammals in which ovarian IT has been characterised [11]. In the interstitial glands in other species, a secondary cell type is seldom described. Instead IT in eutherians [12,21,34,36] and other metatherians [16,20] is described as homogeneous masses or lobules of cells resembling the morphology of the large interstitial cells in the koala. In the rabbit and guinea pig, two interstitial cell types are described, however they are of different developmental origin and occur as separate homogeneous structures [13]. Primary interstitial cells arise independently within the stroma during foetal or neonatal development, appearing as scattered groups of lipid-containing cells [11,13]. Secondary IT develops later, deriving exclusively from luteinised thecal cells of atretic follicles and forming discrete glandular masses that constitute most of the adult IT [13]. Thus, although two interstitial cell types exist in these species, they represent sequential developmental phases and occur in spatially distinct, uniform populations. This contrasts with the koala, in which two morphologically distinct interstitial cell types are interspersed within each focus [11]. This unique conformation raises interesting questions about the potential origins and functional compartmentalisation of IT in the koala. However, such interpretations must be made cautiously given the lack of correspondence with other species. Despite their interspersed structure, large interstitial cells may correspond to luteinised, thecal-derived cells, while small interstitial cells may represent an early stromal-derived population [13]. However, Pagliarani et al. [5] opposed such a conclusion and the existence of a primary stromal-derived interstitial component in the koala, noting that IT was entirely absent in 6-month-old joeys and first appeared only after follicular growth commenced. Pagliarani, et al. [5] proposed that koala IT is formed predominantly through the luteinisation of follicular cells, consistent with a secondary rather than primary origin. Considering this, any correspondence between the two IT cell types in the koala and the primary–secondary dichotomy described in other species must remain tentative.

The heterogeneous composition of interstitial foci in the koala ovary is, however, comparable to the composition of corpora lutea in the species, in which large and small luteal cells are described [5]. The large interstitial cells share close morphological similarity with granulosa-derived large luteal cells, including their polygonal shape, enlarged spherical nuclei and variably vacuolated cytoplasm containing lipid inclusions. Whereas the small interstitial cells resemble theca-derived small luteal cells with their strongly eosinophilic cytoplasm and dense nuclear chromatin, although they lack the cytoplasmic vacuolation that characterises small luteal cells [5]. These parallels suggest that IT and luteal tissue may arise through related luteinisation pathways. Pagliarani et al. [5] described corpus luteum formation as occurring through hypertrophy and luteinisation of granulosa cells to form the large luteal population, accompanied by differentiation of theca interna cells into the small luteal population and invasion of connective tissue to occupy the former follicular cavity.

Immunohistochemistry of interstitial cells in the koala ovary provided preliminary support for their potential capacity for steroidogenesis. It was interpreted from their morphology that the large interstitial cells displayed a distinct steroidogenic phenotype compared to the small interstitial cells. However, immunolocalisation indicated that the latter exhibits a broader range of steroidogenic markers. Small interstitial cells showed the most complete and diverse steroidogenic immunophenotype of all cells in the ovary across reproductive phases, with coexpression of HSD3B2, HSD17B1 and aromatase. During interoestrous, proliferative and luteal phases, HSD3B2 and HSD17B1 immunoreactivity remained moderate to strong, while aromatase maintained mild to moderate staining. Moderate to strong FSHR and mild to moderate LHR immunoreactivity in small interstitial cells during interoestrus and the proliferative phase suggest gonadotropin responsiveness, which provides evidence for their steroidogenic capacity [25,38]. Although not all enzymes involved in ovarian steroidogenesis were assessed in this study, the concurrent expression of HSD3B2, HSD17B1 and aromatase in small interstitial cells provides strong evidence for the presence of steroidogenic pathways within this compartment [27]. In combination, these enzymes indicate the capacity for the sequential conversion of pregnenolone to progesterone (HSD3B2), androstenedione to testosterone and oestrone to oestradiol (HSD17B1), and androgens to oestrogens (aromatase) in response to gonadotropin signalling [39]. This coexpression, therefore, indicates that the small interstitial cells possess the enzymatic repertoire required for the synthesis and local metabolism of multiple steroid classes within the ovarian cortex [39]. However, as this evidence is derived from immunolocalisation alone, it reflects steroidogenic capacity rather than confirmed steroid output, and functional activity within this compartment remains to be demonstrated. The expression of enzymes involved in early steroidogenesis for both progesterone and androgen synthesis including P450scc, HSD3B and CYP17A1 is commonly described in ovarian IT, such as in the brushtail possum [16,17], grey short-tailed opossum (*Monodelphis domestica*) [40], cat [12] and greater Asiatic yellow bat [21]. These findings are in support of the established view that ovarian IT is primarily androgenic, with a variable capacity for progesterone synthesis [11,37]. However, enzymes involved in oestrogen synthesis such as aromatase and HSD17B1 are rarely expressed in mammalian IT and have not yet been reported as detectable in other species [12,16].

In traditional models of ovarian steroidogenesis, aromatase and HSD17B1 are predominantly localised to granulosa cells, where FSH stimulation drives the aromatisation of androgens to oestrogens and the conversion of oestrone to oestradiol, respectively [39,41]. The results of this study demonstrate coexpression of aromatase, HSD17B1 and FSHR in the small interstitial cells of interoestrous and proliferative phase ovaries. This atypical immunophenotype indicates that these cells possess the enzymatic and gonadotropin-responsive machinery required for oestrogen synthesis and conversion of estrone to oestradiol [39]. While functional activity cannot be confirmed from immunoreactivity alone, this coexpression in small interstitial cells is consistent with the possibility that IT may possess the capacity for follicle-independent oestrogen synthesis [42]. This interpretation is in support of the hypothesis that ovarian IT in the koala may synthesise oestrogens that support maturation of the comparatively large pre-ovulatory follicles in the species, although this remains to be functionally validated [8].

In contrast, large interstitial cells displayed a more limited steroidogenic profile. Across reproductive phases, these cells exhibited only mild to moderate HSD3B2 and HSD17B1 immunoreactivity, primarily negative aromatase and negative to mild gonadotropin receptor expression. This pattern is consistent with a less capable steroidogenic phenotype, more closely resembling the interstitial cells in other mammals [11]. Their reduced expression of downstream enzymes and receptors relative to small interstitial cells suggests that the potential capacity for steroidogenesis in large interstitial cells may be limited to low-level progestogen and androgen synthesis. Mild to moderate immunoreactivity for HSD17B1 in combination with minimal aromatase expression is indicative that large interstitial cells may be less capable of contributing to oestrogen synthesis [41]. These findings support the interpretation that small interstitial cells have a greater capacity for steroidogenesis and may be the principal steroidogenic cell within IT foci; this distinctly contrasts with the morphological analysis, which was suggestive of the large cell type being more steroidogenically capable. This apparent discrepancy between the morphology and immunohistochemical profiles of the two cell types highlights marked heterogeneity within interstitial foci. Such unique phenotypic diversity raises novel questions regarding how the coexistence of distinct cell populations could be functionally interpreted, and how their collective organisation may contribute to the overall role of IT within the ovary. It is possible, although unconfirmed, that the highly vacuolated large interstitial cells contribute lipid precursors to the more enzymatically capable small cells. Although speculative, this heterogeneity also raises the possibility that the two cell types may exhibit a division of labour, conceptually analogous to that described between theca and granulosa cells, in which androgen substrates are produced by the former and subsequently converted to downstream steroids by the latter [41]. However, as this is based on morphology and immunophenotype alone, these interpretations remain speculative and require functional validation, for example, through the isolation of interstitial cell populations and in vitro assessment of steroid hormone production [43].

Immunolocalisation in non-interstitial ovarian cells provided additional validation for antibody specificity and offered further insight into functional compartmentalisation within the koala ovary. Although the classical two-cell, two-gonadotropin model remains a useful conceptual framework for follicular steroidogenesis, the distribution of steroidogenic enzymes and gonadotropin receptors in the koala demonstrates a more complex organisation than predicted by this model [44].

Granulosa cells exhibited mild to moderate aromatase and HSD3B2 immunoreactivity across all reproductive phases, together with variable FSHR and LHR expression. Aromatase and FSHR localisation to granulosa cells is consistent with its expected distribution in mammals and therefore provides internal validation for the antibody [41]. Notably, HSD17B1 was absent from granulosa cells in all phases, despite its typical localisation to these cells in mammals [39]. This absence indicates that granulosa cells in the koala may instead be capable of synthesising estrone through the aromatisation of androgen substrates, while oestradiol synthesis may instead occur predominantly in the small interstitial cells, which demonstrated strong HSD17B1 expression [42].

The theca interna displayed negative to mild aromatase and predominantly mild HSD3B2 staining, with primarily negative LHR expression across the reproductive phases. The lack of LHR immunoreactivity in these cells is notable, as LH responsiveness in the theca interna is a central feature of the classical two-cell model [41]. However, the immunolocalisation patterns in this study indicate only limited detectable LH receptor expression, without allowing for functional inference. The theca externa exhibited mild to moderate HSD3B2 staining and low aromatase immunoreactivity, with consistently mild FSHR and negative to mild LHR expression across phases. Expression of aromatase and the capacity for oestrogen synthesis in thecal cells is not typical of classical models; however, it has been described in a range of mammals including humans [45], rhesus monkeys [46], horses [47], sheep [48] and pigs [49]. The low intensity but consistent staining pattern suggests that the thecal layers may possess limited gonadotropin sensitivity and may participate in minor or intermediary aspects of steroid metabolism, although functional significance cannot be determined from immunolocalisation alone [39].

The germinal epithelium demonstrated mild aromatase, HSD3B2 and FSHR immunoreactivity, with consistent absence of HSD17B1 and LHR across reproductive phases. The detection of low-level steroidogenic enzymes in this layer has been described in several mammals and is often associated with local surface signalling or cortical remodelling rather than de novo steroidogenesis [50,51,52].

Together, these findings indicate that steroidogenic activity in the koala ovary is not confined to a strict granulosa-theca dichotomy. Instead, multiple follicular and surface cell populations exhibit variable expression of steroidogenic enzymes and gonadotropin receptors. This supports a model of ovarian steroidogenesis that is more heterogeneous and functionally compartmentalised. This interpretation aligns with a growing body of comparative evidence demonstrating that the traditional two-cell, two-gonadotropin model oversimplifies the diversity of ovarian steroidogenic pathways across mammals and other vertebrates [41,53,54].

Qualitative variation in immunohistochemical staining intensity was observed across reproductive phases in the koala. However, the small and uneven sample sizes across reproductive phases must be considered and therefore cyclical patterns must be interpreted as preliminary. Staining intensity was strongest in the interoestrous, moderate during the proliferative phase, reduced in the luteal phases, and minimal or absent during lactation. These patterns must be interpreted cautiously given the limited sample size, variability and lack of the precise timing of the phase, although the overall trend was consistent across all markers examined. The reduction in both enzyme and gonadotropin receptor immunoreactivity during luteal and lactational phases is consistent with the general mammalian suppression of follicular endocrine activity in these periods [55,56]. Lactational studies in the koala and other marsupials have supported this mechanism, whereby the suckling stimulus inhibits gonadotropin secretion and prevents the initiation of new follicular waves [29,57]. The stronger interstitial immunoreactivity during interoestrus, together with the pronounced vacuolation of large interstitial cells observed, may represent a preparatory or modulatory phase. During interoestrus, lipid stores and steroidogenic activity may increase following follicular atresia and precede the initiation of subsequent follicular waves [36]. Targeted studies with larger datasets are required to determine whether these preliminary observations reflect true cyclical regulation of IT function in the koala.

It is critical to consider that the interpretations of this study’s findings are constrained by several methodological limitations. Firstly, while immunohistochemistry identifies protein localisation, it does not confirm enzymatic activity or steroidogenic output [58,59]. No hormonal quantification or transcript-level validation was performed, so correlation between protein expression, mRNA abundance, and steroid output cannot be inferred. As a result, the patterns of enzyme and receptor expression identified only indicate a capacity for steroidogenesis within the koala ovary. Accordingly, the absence of functional data means these observations remain preliminary and require confirmation through targeted hormonal and molecular analyses. The application of liquid chromatography–mass spectrometry to in vivo and in vitro samples, particularly when combined with appropriate experimental designs, would allow for discrimination between steroid precursors and biologically active steroids produced by IT [60]. Additionally, the small and uneven sample size, driven by opportunistic specimen availability and limited representation of precisely timed reproductive phases, also restricts the generalisability of these observations. The lack of supporting behavioural and endocrine data necessary to validate reproductive phase classification further constrains the interpretation of phase-associated differences in immunophenotype and may limit the resolution of subtle cyclical changes [5]. While this investigation cautiously interprets cyclical variation in morphology and protein expression, its primary outcome is the demonstration of steroidogenic capacity in the IT across phases. Future investigations incorporating a broader and more evenly distributed representation of reproductive phases validated by behaviour or endocrinology will be necessary to account for inherent inter-individual variability within each phase. Such improvements would facilitate a more accurate characterisation of potential cyclical variation in IT morphology and immunophenotype [26]. Finally, antibody specificity cannot be fully established without marsupial- or koala-validated reagents [61]. Immunohistochemical studies in the koala are limited, therefore investigations necessarily rely on antibodies validated in other species and indirect approaches to assess specificity. These limitations require cautious interpretation of immunolocalisation data in the absence of koala-specific reagents [9,62]. In this study, specificity was addressed indirectly through prior sequence homology assessment, concordance with established cellular localisation patterns in other species, and the use of internal positive and negative tissue controls [32,61]. Accordingly, the results are discussed in terms of steroidogenic capacity rather than confirmed functional activity. Despite these constraints, rigorous optimisation of protocols and reliance on consistent internal controls increases the reliability of the observed staining patterns and interpretations [30,61]. Consequently, this study provides preliminary but robust evidence that can inform and justify future targeted investigations into koala ovarian physiology. Future work incorporating mRNA analyses [17,39], in vivo and in vitro hormone quantification [19,21,36,43] and expanded sampling across reproductive phases will be essential to validate and extend these preliminary observations.

## 5. Conclusions

The combined morphological and immunohistochemical findings of this study provide preliminary evidence that ovarian IT in the koala possesses the molecular components required for steroidogenesis. Two morphologically distinct interstitial cell populations were consistently identified within each focus, a configuration described as unique amongst mammals. The large interstitial cells exhibited a classical steroidogenic phenotype, including vacuolated cytoplasm consistent with lipid mobilisation, while the small interstitial cells lacked such characteristics. In contrast, immunohistochemistry demonstrated that the small interstitial cells expressed the most complete and diverse steroidogenic profile in the koala ovary, while the large cell type exhibited a comparatively limited immunophenotype. This potential functional division between morphologically distinct cell types, combined with the unusual heterogeneity of IT foci, suggests an atypical pattern of steroidogenic compartmentalisation in the koala. Immunohistochemical staining for these markers was strongest during interoestrus, moderate in the proliferative phase, reduced in the luteal phase and minimal in lactating koalas. This suggests, but does not confirm, a cyclical pattern in which IT may be primed for steroidogenesis outside periods of active reproductive function. However, the qualitative measurements and small sample size warrants cautious interpretation. These findings provide new insight into the unique ovarian physiology of the koala and highlight the need for further research to determine the functional steroidogenic output of IT, its regulation across reproductive phases and its broader physiological significance. While the results of this investigation provide evidence for the steroidogenic capacity of IT in the koala ovary, the absence of direct functional measures precludes confirmation of steroidogenic activity. As such, conclusions regarding the potential functional role of IT are necessarily inferential and based on morphological and immunohistochemical evidence alone. Further studies incorporating endocrine measurements and gene expression analyses will be required to validate the functional significance of these observations and to determine the extent to which IT contributes to ovarian steroidogenesis in this species.

## Figures and Tables

**Table 1:** Morphological characteristics of the ovary and uterus used to classify koala reproductive phases (interoestrus, proliferative, luteal). Lactational anoestrus classification was determined based on presentation with pouch young and/or lactating mammary glands. Adapted from Johnston [8] and Pagliarani [9].

Reproductive Phase	Corpus luteum (CL)	Pre-Ovulatory Graafian Follicle > 4 mm Diameter	Uterine Morphology
Interoestrus	No	No	Minimal or undeveloped and narrow endometrial glandular tissue characterised by sparsely ciliated, simple cuboidal or columnar epithelia.
			Endometrium slightly thinner (≈0.75 mm) on average relative to myometrium (≈1.15 mm).
Proliferative	No	Yes	Significantly thickened endometrium characterised by highly vascular submucosa containing dense, open endometrial glandular tissue.
			Endometrial glandular lumina lined with hyperplastic, simple ciliated columnar epithelial cells, interspersed with goblet cells.
			Endometrium significantly thicker (≈2.36 mm) on average relative to myometrium (≈1.28 mm).
Luteal	Yes	No	Endometrial tissue characterised by a bi-layered appearance composed of basal and superficial layers, differentiated by increased density of glandular endometrial tissue in the superficial layer.
			Endometrium significantly thicker (≈3.8 mm) on average relative to myometrium (≈0.4 mm).
			Post-luteal uteri characterised by degenerative morphology and luminal sloughing.

**Table 2:** Primary antibodies used for immunohistochemistry in koala ovarian tissue, showing target, clonality, host species, predicted homology with koala proteins, subcellular localisation, and working dilution.

Antibody	Product Number	Clonality	Host Species	Homology with Koala Protein	Predicated Localisation	Working Dilution	Antigen Retrieval Method
Aromatase (CYP19A1)	PA5-109235	Polyclonal	Rabbit	90.0%	Cytoplasm	1:400	Low pH
HSD3B2	PA5-106895	Polyclonal	Rabbit	68.6%	Cytoplasm	1:1200	Low pH
HSD17B1	PA5-42058	Polyclonal	Rabbit	78.0%	Cytoplasm	1:200	Low pH
LHR	PA5-79598	Polyclonal	Rabbit	88.2%	Membrane	1:400	Low pH
FSHR	PA5-99424	Polyclonal	Rabbit	72.6%	Membrane	1:200	Low pH

**Table 3:** Immunohistochemical staining intensity of different ovarian cell types across specific reproductive phases in the koala. Values represent the modal staining category for each antibody and cell type based on all examined samples. Staining intensity was classified semi-quantitatively as negative (−), mild (+), moderate (++), or strong (+++). G = granulosa cells; TI = theca interna; TE = theca externa; LITC = large interstitial cells; SITC = small interstitial cells; GE = germinal epithelium.

Reproductive Phase	Cell Type	LHR	FSHR	Aromatase	HSD3B2	HSD17B1
Interoestrus	G	++	+/++	+	++	−
	TI	−	+	−/+	+	−
	TE	−/+	+	+	+/++	−
	LITC	+	+	+	+	++
	SITC	++	+++	++	++/+++	+++
	GE	−	++	+/++	++	−
Proliferative	G	+/++	+	+	+	−
	TI	−/+	+	−/+	−/+	−
	TE	−/+	+	−	+/++	−
	LITC	+	+	−	+	+/++
	SITC	+/++	+	+	++/+++	++
	GE	−	+	+/++	+/++	−
Luteal	G	−/++	−/+	+/++	+/++	−
	TI	−	−/+	+	−/+	−
	TE	−/+	−/+	+	+	−
	LITC	−/+	−/+	−/+	+	−/++
	SITC	+/++	−/+	+/++	++/+++	−/++
	GE	−	−/+	−/++	+/+++	−
Lactational anoestrus	G	+	+	−/+	+	−
	TI	−	+	−	−/++	−
	TE	−	+/++	−	++	−
	LITC	−	+	−	−	+
	SITC	−	+	−	+	+
	GE	−	−	−	−/+	−

## Data Availability

The raw data supporting the conclusions of this article will be made available by the authors on request.

## Supplementary Materials

The following supporting information can be downloaded at: https://www.mdpi.com/article/10.3390/biology15010047/s1, Figure S1: No primary antibody- and negative tissue controls used for immunohistochemical validation of LHR, FSHR, aromatase, HSD3B2, HSD17B1 antibodies. (**A**) Koala ovary used a no primary antibody control tissue for anti-LHR showing negative staining. (**B**) Koala colon used as negative tissue control for anti-LHR showing negative staining. (**C**) Koala ovary used a no primary antibody control tissue for anti-FSHR showing negative staining. (**D**) Koala lung used as negative tissue control for anti-FSHR showing negative staining. (**E**) Koala ovary used a no primary antibody control tissue for anti-aromatase showing negative staining. (**F**) Koala lung used as negative tissue control for anti-aromatase showing negative staining. (**G**) Koala ovary used a no primary antibody control tissue for anti-HSD3B2 showing negative staining. (**H**) Koala lung used as negative tissue control for anti-HSD3B2 showing negative staining. (**I**) Koala ovary used a no primary antibody control tissue for anti-HSD17B1 showing negative staining. (**J**) Koala skeletal muscle used as negative tissue control for anti-HSD17B1 showing negative staining. Bar = 1mm (**A**,**C**,**E**,**G**,**I**). Bar = 50 μm (**B**,**D**,**F**,**H**,**J**).

## Abbreviations

The following abbreviations are used in this manuscript:

IT	Interstitial tissue
LHR	Luteinising hormone receptor
CYP19A1	Cytochrome P450 family 19 subfamily A member 1
FSHR	Follicle-stimulating hormone receptor
HSD3B2	3β-hydroxysteroid dehydrogenase/Δ5à4-isomerase type 2
HSD17B1	17β-hydroxysteroid dehydrogenase type 1
G	Granulosa
TE	Theca externa
TI	Theca interna
LITC	Large interstitial cells
SITC	Small interstitial cells
GE	Germinal epithelium
HRP	Horseradish peroxidase
NBF	Neutral-buffered formalin
BLASTp	Basic protein local alignment search tool
