# Supplementary figures and images for: Steroidogenic Capacity of Ovarian Interstitial Tissue in the Koala (Phascolarctos cinereus): Morphological and Immunohistochemical Evidence
Source: Biology (Basel). 2025 Dec 27;15(1):47. doi: 10.3390/biology15010047 (PMC12785116; doi:10.3390/biology15010047)

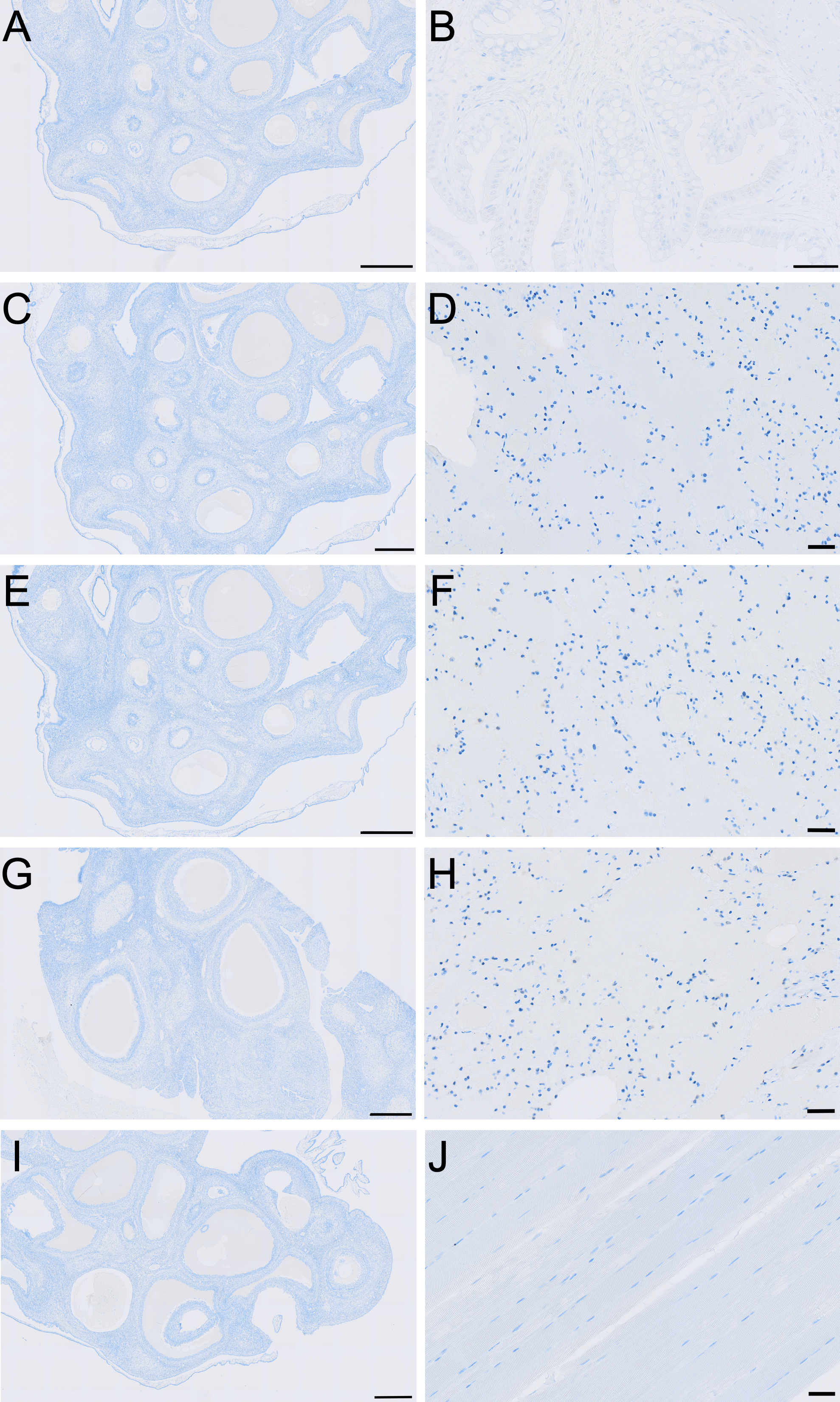

Supplement: Supplementary file 1 [file biology-15-00047-s001.zip › Figure S1.png]
